# Setting up and managing the largest COVID-19 mass vaccination center in Lombardy, Italy

**DOI:** 10.3389/fpubh.2023.1290350

**Published:** 2023-11-17

**Authors:** Jessica Graziella Calegari, Alberto Bisesti, Silvia Pazzaglia, Simone Gambazza, Filippo Binda, Martina Bruno, Laura Chiappa, Alessandra Piatti, Navpreet Tiwana, Matteo Letzgus, Silvana Castaldi, Marcello Sottocorno, Dario Laquintana

**Affiliations:** ^1^Healthcare Professions Department, Fondazione IRCCS Ca’ Granda Ospedale Maggiore Policlinico, Milan, Italy; ^2^Hospital Medical Direction, Fondazione IRCCS Ca’ Granda Ospedale Maggiore Policlinico, Milan, Italy; ^3^Department of Biomedical Sciences for Health, University of Milan, Milan, Italy; ^4^Quality Unit, Fondazione IRCCS Ca’ Granda Ospedale Maggiore Policlinico, Milan, Italy; ^5^Department of Hospital Pharmacy, Fondazione IRCCS Ca’ Granda Ospedale Maggiore Policlinico, Milan, Italy

**Keywords:** vaccine, COVID-19, mass vaccination center, vaccination experience, organizational layout

## Abstract

**Background:**

The rapid global spread of severe acute respiratory syndrome coronavirus (SARS-CoV-2) was met with the rollout of vaccination campaigns at mass vaccination centers. The Palazzo delle Scintille, Milan, was designated by the Lombardy regional administration as a vaccination site with the target of processing about 9,000 users daily.

**Methods:**

For this observational study, we compared data on vaccinations delivered at the Palazzo delle Scintille with coronavirus disease (COVID-19)-related regional data.

**Results:**

Between 25 April 2021 and 28 February 2023, a total of 1,885,822 COVID-19 doses were administered; the mean hourly rate was 289 (247.2), the mean daily rate was 3185.5 (3104.5), the mean user age was 49.5 years (10.7). The Comirnaty vaccine (Pfizer-BioNTech) was most often given (1,072,030/1,885,822; 56.8%). Between 4 December 2021 and 15 January 2022, the daily dose rate was above the maximum daily capacity set by the regional administration.

**Conclusion:**

The trend for daily dose rates administered at the Palazzo delle Scintille center was in line with COVID-19-related regional data. The center played a major role in the regional mass vaccination campaign.

## 1 Introduction

Highly infectious, often fatal, and with the potential for rapid spread, severe acute respiratory syndrome coronavirus 2 (SARS-CoV-2) was met with mass vaccination campaigns to curb the spread of coronavirus disease (COVID-19) (1). Lombardy was the first region in Europe to be struck by the disease; (2, 3) the initial transmission rates were so high that area hospitals were soon overloaded with severely ill patients (4, 5). In an effort to lighten the burden on hospital services, alternative venues were designated as mass vaccination centers (MVC) (1, 6, 7). At the start of the COVID-19 pandemic, the Lombardy regional administration relied on existing community-based vaccination clinics and fully equipped hospitals working within the Italian national health service which, however, were found unable to sustain delivery of high-volume vaccination, maintain stock, give additional shots of certain types of vaccines, and ensure the cold chain (1, 8). The regional administration then designated large public venues (e.g., exhibition spaces) to be converted into MVCs and mandated 65 community and research hospitals in Lombardy (9) to manage 56 mass vaccination sites (10).

The Palazzo delle Scintille, located in Milan, the largest of the region’s vaccination sites, was set a daily target of 9,936 vaccinations when operating at maximum capacity (9–11). The Fondazione IRCCS Ca′ Granda Ospedale Maggiore Policlinico was mandated to manage the MVC, under the responsibility of the hospital’s administrative board of healthcare professionals, which was assigned the organization and the management of the site’s operations. The Palazzo delle Scintille MVC was designed according to information provided by the Lombardy regional administration and based on a model studied in the Fiera Milano City pavilion (9, 12). The influenza vaccine was administered to eligible persons (13) jointly with the COVID-19 vaccine during two flu vaccination campaigns (2021/2022 and 2022/2023 seasons) promoted by the regional administration to foster flu vaccination adherence.

The aim of the present observational study was: (1) to quantify the longitudinal activity of the MVC expressed as COVID-19 vaccine dose rates by comparing the center’s rates with COVID-19-related regional data; (2) to evaluate the organizational and managerial operations at the MVC; and (3) to describe the administration of influenza vaccination.

## 2 Materials and methods

### 2.1 Organization of space and user flow inside the Palazzo delle Scintille MVC

**Figure 1:**
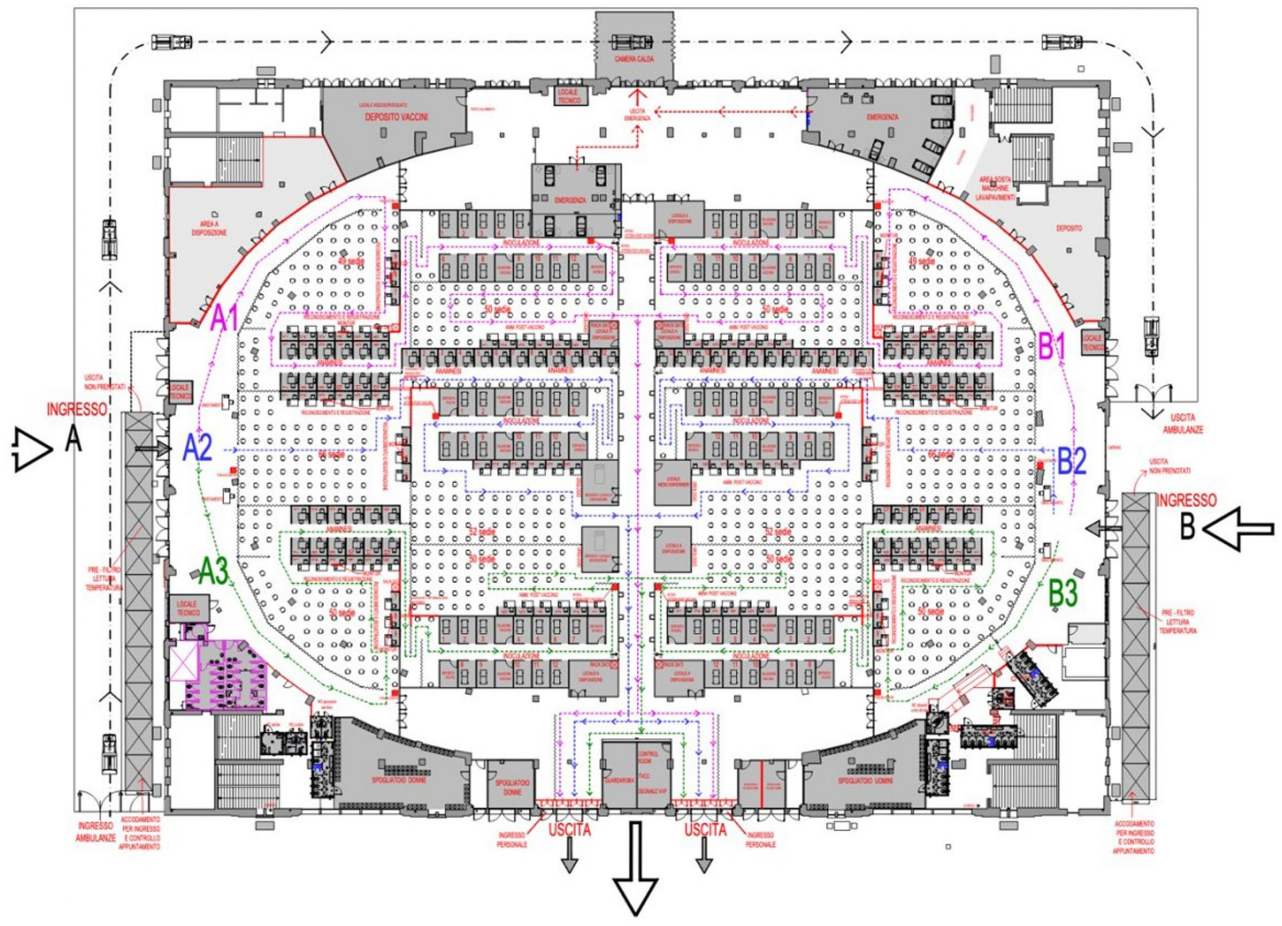
Floor plan and users flow. The arrows indicate the direction of user flow inside the Palazzo delle Scintille MVC.

The Palazzo delle Scintille was selected because of its convenient location and space capacity. The site is served by surface and underground public transport, making it easy to reach from other parts of Milan or the metropolitan area. The center measures 15,500 square meters in floor area, which was subdivided into stations in linear sequence along which users advanced (check-in, evaluation, vaccine administration, observation), similar to the pathway layout of other large-scale vaccination centers in Italy and around the world (1, 12, 14–19). The center was divided into two identical areas (A and B), each with its own entrance. Each area was subdivided into three modules with 24 stations: 12 for evaluation and 12 for vaccination, for a total of 144 stations (Figure 1). Appointments could be made via the Poste Italiane web site, where users could check waiting times and center opening times. Users could also come on a walk-in basis without booking an appointment.

Based on estimated user flow projection from the data recorded on the Poste Italiane platform, the MCV management checked with the pharmacy about the daily availability of doses before scheduling the number of vaccination lines (i.e., evaluation and vaccination by one physician and one nurse at two stations) and modules to be activated each week.

### 2.2 Staff management and training

The center was staffed by physicians, nurses, and pharmacists for vaccine stockage and administration, as well as support staff, including clerical workers, volunteers, and National Guard members who assisted in directing user flow and data collection. The hospital administrative board oversaw the work of the head manager, the head pharmacist, the chief physician, and the chief nurse employed at our institution, as well as staff from community (ASST Santi Paolo e Carlo, Rhodense, Pini-Trauma Center, Fatebenefratelli-Sacco) and research hospitals (Fondazione IRCCS Istituto Neurologico Carlo Besta, Fondazione IRCCS Istituto Nazionale dei Tumori) located in the Milan metropolitan area, which collaborated during peak service times.

Given the number of people involved in widely diverse tasks, staff turnover, and urgent recruitment, staff training was conducted online, with the aid of operative training in specific stages of operation, roles, and responsibility of each staff member, as well as instruction on electronic data entry. The operative training was updated by integrating information and guidelines issued by national and regional governments and the Italian Medicines Agency to ensure staff received accurate and timely information.

### 2.3 Statistical analysis

Data on daily rates of COVID-19 and influenza vaccination administered at the center were collected via the Poste Italiane website services granted the Lombardy regional administration to support the mass vaccination campaign. The data on the number of COVID-19 vaccinations and SARS-CoV-2-positive swabs recorded for Lombardy were downloaded from the GitHub software platform (20). Data on persons who were vaccinated at the center between 25 April 2021 and 28 February 2023, opening and closing dates, respectively, were exported into Excel and processed. Data are expressed as the mean and standard deviation (SD) or total frequency and percent (%). Longitudinal trends for dose delivery rates at the center and at other vaccination centers in Lombardy and the number of COVID-19 cases recorded for Lombardy were obtained by nonparametric smoothing. Two-sided chi-square test was used to compare the yearly influenza vaccination rates. Statistical analysis was performed using Open Source R software (21).

## 3 Results

### 3.1 COVID-19 vaccination delivery volume

**Figure 2:**
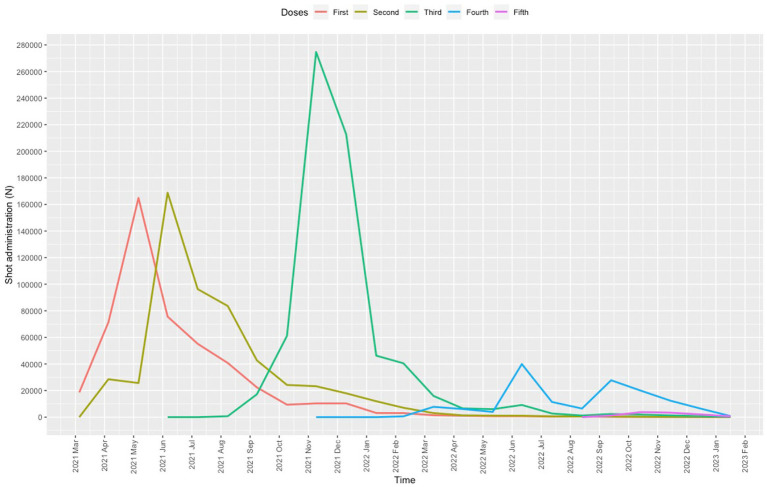
Volume of COVID-19 vaccinations stratified by dose (first to fifth).

Between 25 April 2021 and 28 February 2023, a total of 1,885,822 COVID-19 vaccinations were administered, 957,442 (50.8%) of which were given to males; the hourly dose rate was 289 (247.2) and the daily dose rate was 3185.5 (3104.5) (range, 53 on 22 February 2022 to 12,826 on 22 July 2021). The mean user age was 49.5 (10.7) years (range, 5–108). A total of 139,585 (7.4%) vaccinations were administered to children (age < 18 years) and 431,024 (22.9%) to older adults (age ≥ 60 years). The first dose was given to 490,695 users, the second to 539,049, the third to 701,619, the fourth to 143,477, and the fifth to 10,982 (Figure 2).

**Figure 3:**
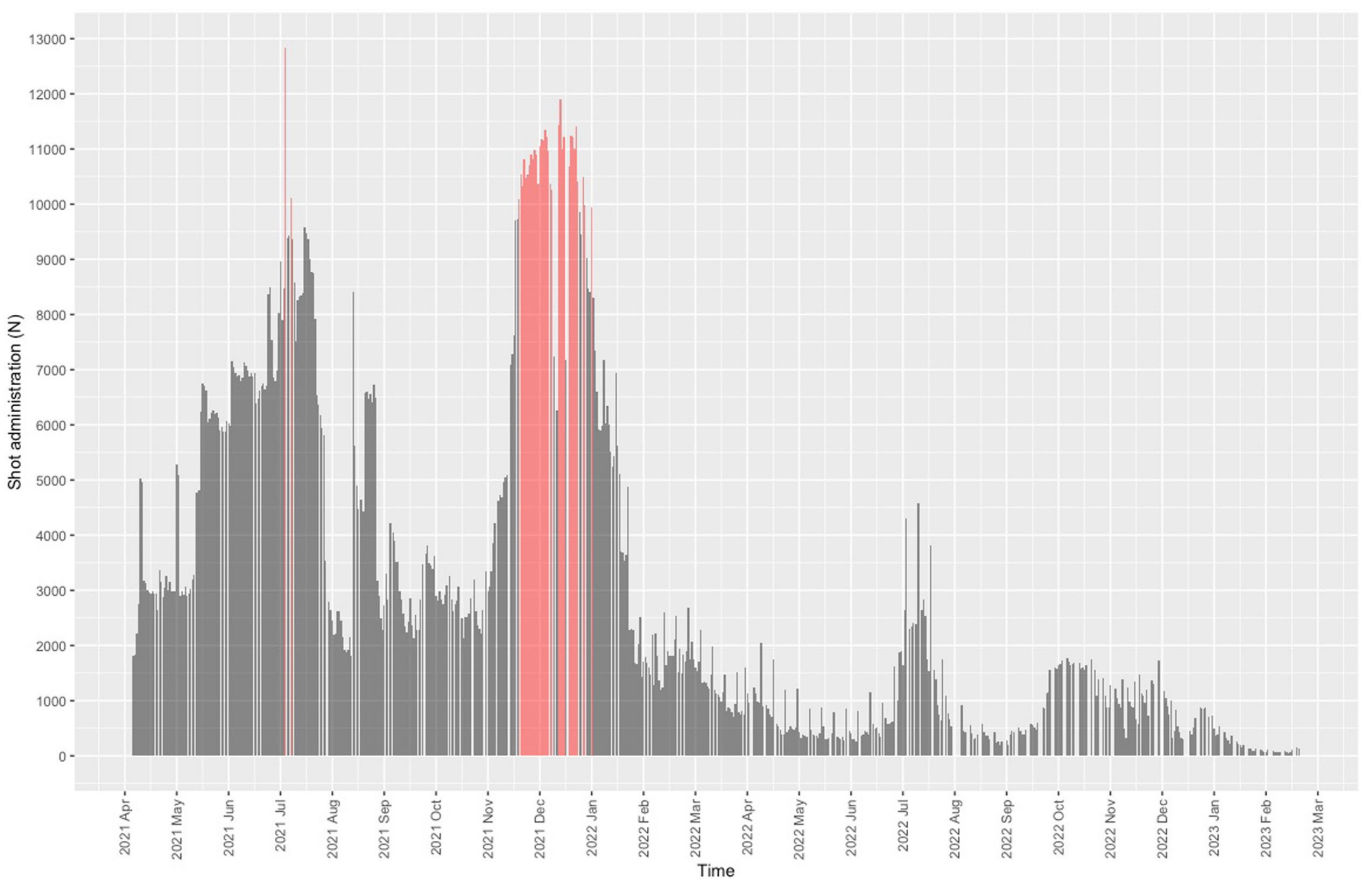
User frequency. Red bars denote the days when the center operated above maximum capacity.

Starting on 4 December 2021, the center began operating beyond the capacity set by the Lombardy administration (9,936 daily doses), except for two days during the summer months (Figure 3). The mean daily dose rate recorded for December 2021 was 10,276 (3.35% first dose, 7.55% second dose, 89.10% third dose). The center worked over capacity on 35 days.

The vaccine most often given was Comirnaty (Pfizer-BioNTech; 1,072,030/1,885,822, 56.8%), followed by Spikevax (Moderna; 629,435/1,885,822, 33.4%), Vaxzevria (AstraZeneca; 59,000/1,885,822, 3.1%), Janssen (Johnson & Johnson; 11,070/1,885,822, 0.6%), Comirnaty Pediatric (Pfizer-BioNTech; 10,921/1,885,822, 0.6%), and Nuvaxovid (Novavax; 2180/1,885,822, 0.1%). Two bivalent vaccines, Comirnaty Plus 10 and Plus B.A. 4–5 and Spikevax B.A. 4–5 (84,131/1,885,822, 4.5% and 6604/1,885,822, 0.3%, respectively), were administered as additional (booster) vaccinations to users aged 12 years and older. Starting in January 2023, Original/Omicron BA.4–5, the bivalent pediatric formulation of Comirnaty, was authorized for use in children aged 5–11 years and given to 77 children (22).

**Figure 4:**
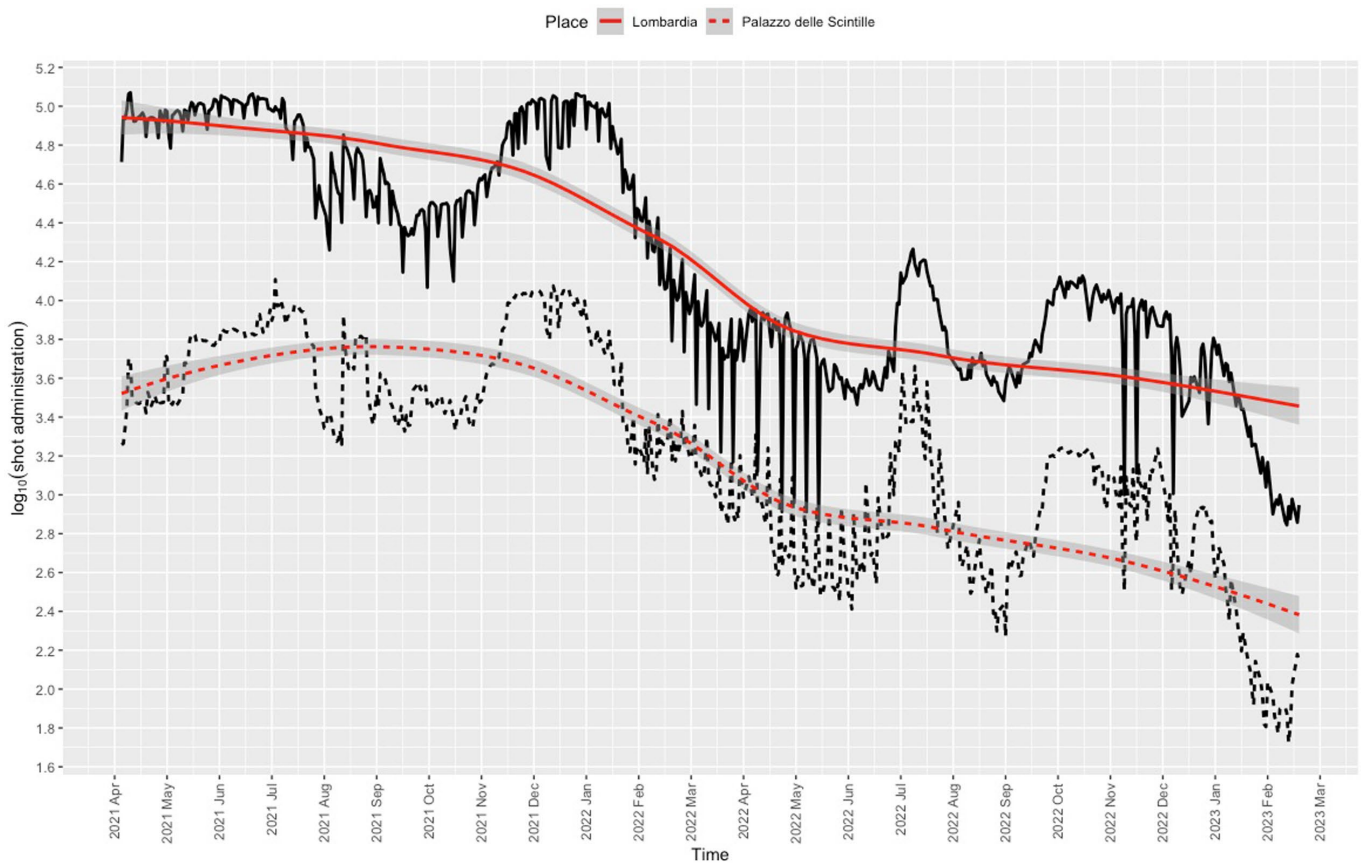
Frequency of COVID-19 vaccination recorded for Lombardy (solid line) and for the Palazzo delle Scintille MVC (dotted line). Red lines denote nonparametric locally weighted running line smoother with 95% confidence interval bands.

**Figure 5:**
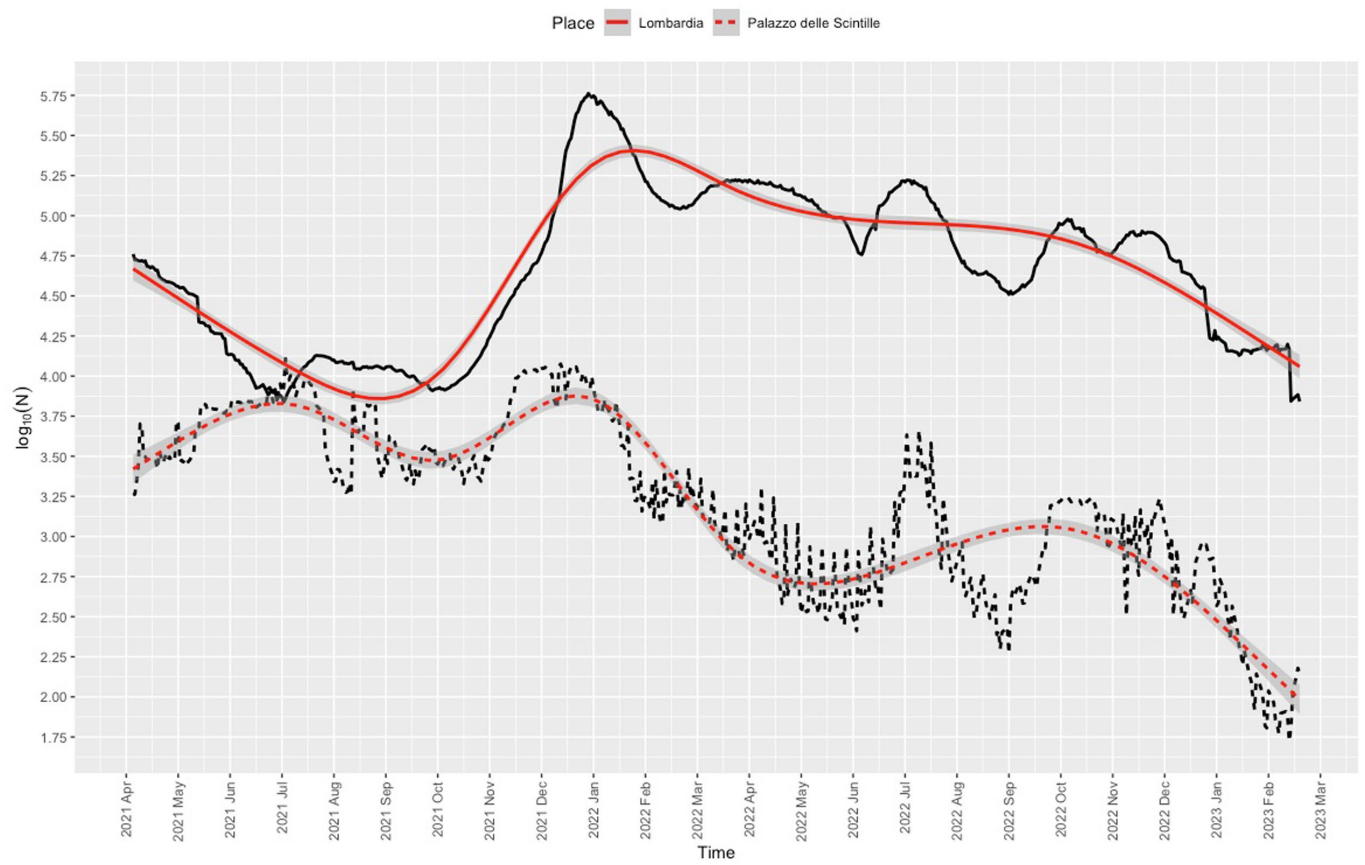
Frequency of COVID-19 positive cases in Lombardy (solid line) and frequency of COVID-19 vaccinations administered at the Palazzo delle Scintille MVC (dotted line). Red lines denote nonparametric smoothing using restricted cubic splines with 8 knots, with 95% confidence interval bands.

Nearly all users (1,848,527/1,885,822, 98%) were residents of Lombardy, 95% (1,755,708/1,848,527) were residents of the province of Milan, and 72.9% (1,279,819/1,755,708,) were residents of the city of Milan. The proportion of vaccinations given at the center was 8.4% (1,885,819/22,427,453) of the total administered in Lombardy. The daily dose rate was in line with the rates recorded for other vaccination center in Lombardy (Figure 4). The trend for the daily dose rate recorded for the center compared to the trend for the number of COVID-19 cases in Lombardy is shown in Figure 5.

### 3.2 Influenza vaccination delivery volume

During the 2021 winter flu season, a total of 49,045 influenza vaccinations were administered to persons aged 70.2 (7.8) years, on average (range, 3–104), making up 2.6% (49,045/1,842,201) of the influenza vaccinations administered in Lombardy between October 2021 and February 2022. The vast majority (79.7%) were aged 60 years or older, while children (<18 years) accounted for only 0.1% (32/49,045).

During the 2022 winter flu season, 30,688 influenza vaccinations were administered to persons aged 59 (8.8) years, on average (range, 1–108); 69.8% were aged 60 years or older and 1.6% aged under 18 years, with 305 (1%) under age 12 years. Table 1 presents the yearly dose volumes for 2021 and 2022. The rate was 8,527 for October 2021 (versus 82,278 COVID-19 vaccinations) and 12,124 for October 2022 (versus 32,048 COVID-19 vaccinations). The proportion of influenza vaccinations was 9.4% (85,527/90,805) for October 2021 and 27.45% (12,124/44,172) for October 2022.

**Table 1:** Monthly influenza vaccination rates for 2021 and 2022.

	2021	2022	*p*-value
October	8573 (17.5)	12124 (39.5)	<0.001
November	22517 (45.9)	10094 (32.9)	<0.001
December	16752 (34.2)	7106 (23.2)	<0.001
January	1188 (2.4)	1321 (4.3)	<0.001
February	15 (0.03%)	43 (0.1%)	<0.001
Total	49045	30688	

Data are presented as counts and percentage (%).

### 3.3 Staff

A total of 6,500 physicians and nurses were credentialed via the Poste Italiane web site for administering vaccinations at the center. Physicians and nurses from eight community and research hospitals in the Milan metropolitan area, primary care providers, freelance healthcare providers, and volunteer professionals participated in the vaccine campaign.

## 4 Discussion

The Palazzo delle Scintille MVC worked beyond capacity of the daily dose rate set by the Lombardy administration during December 2021 and early January 2022. User flow gradually decreased starting in February 2022, reflecting the general decline in adherence to the COVID-19 vaccination campaign in Lombardy. The center offered both influenza and COVID-19 vaccination during the 2021 and 2022 winter campaigns; the number of vaccinations was higher for October 2022 than for October 2021.

Across the globe, MVCs responded adequately to the COVID-19 public health emergency (1, 8, 18, 23) by providing infrastructure and space at large public venues converted into high-throughput vaccination sites. MVCs supported campaigns for population-wide vaccination (14, 24–26). User flow design was based on previous queueing process models of areas for user registration, evaluation, vaccination, and observation (12, 14, 16–18). The modular layout allowed for sector deployment according to daily user flow rate and for flexible capacity utilization (12, 14, 19).

During peak user flow periods, people who had booked in advance were given priority and joined a separate queue at the entrance of the center for their appointment. This meant that the number of vaccination lines and sectors had to be adjusted as needed to meet demands within the center’s operating hours and doses available. Finding additional resources among doctors and nurses was the greatest challenge: the majority came to work at the center on their days off. They were paid for their time working at the center.

The pandemic struck Lombardy more severely than elsewhere in Europe (2, 4). The first COVID-19 outbreak occurred in February 2020, followed by five waves throughout the country: the first occurred in spring (March–May 2020) with an overall death toll of 33,606; the second in fall/winter (October 2020 through January 2021); the third from February to May 2021; the fourth from June 2021 to October 2021, and the fifth and final from November 2021 to February 2022 (27). The Italian COVID-19 vaccination campaign was similar to that of other European Union countries, in which the target population was differentiated by social category. Initially, priority was given to health and social workers, nursing home residents and staff, people aged 80 years or more, and those aged between 60 and 79 years and living with chronic disease (phase-1 campaign). The campaign was extended to law enforcement, teachers and school staff, pharmacists, veterinarians (phase-2 campaign), then to people with comorbidities aged less than 60 years, and ultimately to the remaining population (phase-3 campaign) (17). After 2,853,287 doses had been administered in Lombardy, the center began operation to support the phase 3 campaign during the third pandemic wave. At that time, the vaccinable population over 5 years of age was 9,904,997 and the people who completed the vaccination cycle with two doses or single dose were 876,596.

The target daily dose rate at the center was 9,936. Between 4 December 2021 and 15 January 2022 the rate was above capacity probably because users could get a vaccination at the center on a first-come-first-served walk-in basis. The center was busiest when users came for additional vaccinations (28, 29) and during the Christmas holidays. To meet the heightened demand, an organizational model including fast tracks was adopted to shorten service times: users could be evaluated and vaccinated at the same station, thus shortening waiting times between user areas, as suggested in a study by Smith et al. (18) and observation of other MVCs in Italy (12, 16).

Based on this model, users were categorized according to the dose they came to receive. Users who came to the center for their first or second dose or presented with major medical issues (e.g., known allergy to a vaccine component) underwent medical evaluation prior to vaccination by a physician, whereas the remaining users were administered their vaccination by a nurse. This reorganization reduced processing time in response to user experience and dissatisfaction with long waiting times (18, 30).

The daily dose trend appears to partially follow that of SARS-CoV-2 positive cases reported for Lombardy. Starting from the opening of the center through to the deployment of services for booster vaccination during the COVID-19 vaccination campaign (28), parallel trends can be noted for the number of vaccinations administered and the number of COVID-19 positive cases recorded for Lombardy (September 2021). The decline in vaccinations followed the decrease in the number of positive cases in Lombardy starting in February 2022. Vaccination rates peaked twice: once between December 2021 and January 2022 when booster shots became available for children under age 18 years and adolescents aged 12 to 15; (31) then again when possession of the so-called Green Pass COVID-19 became mandatory (32) from the summer to the autumn of 2022, during which a second booster was recommended for adults ≥60 years, (33, 34) and fines were issued to anyone who did not complete the vaccination cycle, (35) as well as the deployment of mRNA vaccine (bivalent Original/Omicron BA.1). The number of SARS-CoV-2-positive cases in Lombardy and the daily dose rates declined after 1 November 2022 when vaccination was no longer mandatory (36). However, media attention to, expert opinion on, and government promotion of the vaccination campaign may have nudged people to book their vaccination at the center, especially because of the expected increase in transmission of COVID-19 infection. The similarity between the curves might also be explained by the timing of vaccination against respiratory virus.

The total number of influenza vaccinations at the center was lower for 2022 and 2023 than for 2021 and 2022, though more people were vaccinated in October 2022 than in 2021. The difference may be explained by the recommendations issued by the Ministry of Health to move forward to October that year’s influenza vaccination campaign (37). Together with the decline in the number of COVID-19 vaccinations, the number of influenza vaccinations in the following months was lower than in previous years because it could be received at the same visit for the COVID-19 vaccination, except for open day events sponsored by the regional administration to specifically promote influenza vaccination campaigns. This decrease in the 2022–2023 flu vaccination rate occurred although flu virus circulation the previous year in Europe (2021–2022 season) was higher than that after the onset of the COVID-19 pandemic in 2020. Moreover, this unusually late onset of the 2021–2022 influenza season might have been influenced by the COVID-19 pandemic. Measures implemented during the winter could have delayed the onset when COVID-19 restrictions were lifted (38). The influenza incidence was nevertheless significantly lower in the 2021–2022 flu season than the pre-pandemic levels. The decision to combine the administration of influenza vaccines and COVID-19 booster vaccines may have been an effective strategy to increase immunization coverage (39). As stated by Domnich et al., public acceptance of vaccination with both vaccines simultaneously was relatively low in Italy, despite the reported advantages of co-administration (40).

Over 7,000 persons were involved in keeping the center running. Clinical staff worked daily shifts of 12, 10 or 6 h depending on daily bookings and user frequency at 144 stations during full operation. Volunteers were vital in relieving clinical staff of nonclinical tasks such as directing use flow and providing help and assistance. The importance of engagement by nonclinical staff in the daily operation of a MVC resides in the increased need for recruiting clinical staff for patient management in hospitals and vaccination campaigns (14–16, 19, 41).

### 4.1 Strengths and limitations

Set within a regional, national, and international context, the set up and management of the Palazzo delle Scintille COVID-19 MVC provides an example for emergency preparation in the prompt delivery of health and medical services. A limitation to the present study is that we were unable to collect adequate data on the human resources employed in the center’s daily operations; therefore, we cannot draw correlations between daily frequency and constraints on staff availability. Fast vaccination tracks were deployed during the period when the center operated above capacity in an effort to optimize workflow and respond to use dissatisfaction. Unfortunately, we were unable to measure the impact that the organizational model had on these variables due to the lack of data systems for tracking the number of resources involved and the vaccination waiting times.

## 5 Conclusion

Between December 2021 and January 2022 the Palazzo delle Scintille MVC operated above capacity set by the regional administration. User frequency reflected the evolution of regulations concerning COVID-19 vaccinations. The experience gained from setting up and managing the Palazzo delle Scintille MVC can inform the establishment of similar MVCs in other regions or countries.

## Data availability statement

The raw data supporting the conclusions of this article will be made available by the authors, without undue reservation.

## Author contributions

JGC: Conceptualization, Data curation, Investigation, Software, Writing – original draft. AB: Conceptualization, Resources, Writing – review & editing. SP: Resources, Writing – review & editing. SG: Formal analysis, Methodology, Visualization, Writing – original draft. FB: Investigation, Writing – original draft. MB: Investigation, Writing – review & editing. LC: Resources, Supervision, Writing – review & editing. AP: Supervision, Writing – review & editing. NT: Supervision, Writing – review & editing. ML: Supervision, Writing – review & editing. SC: Writing – review & editing. MS: Supervision, Writing – review & editing. DL: Conceptualization, Resources, Supervision, Writing – review & editing.
